# Supplementary material for: Identification and Testing of Antidermatophytic Oxaborole-6-Benzene Sulphonamide Derivative (OXBS) from Streptomyces atrovirens KM192347 Isolated from Soil
Source: Antibiotics (Basel). 2020 Apr 13;9(4):176. doi: 10.3390/antibiotics9040176 (PMC7235740; doi:10.3390/antibiotics9040176)
Supplement: Supplementary file 1 [file antibiotics-09-00176-s001.pdf]

antibiotics

MDPI

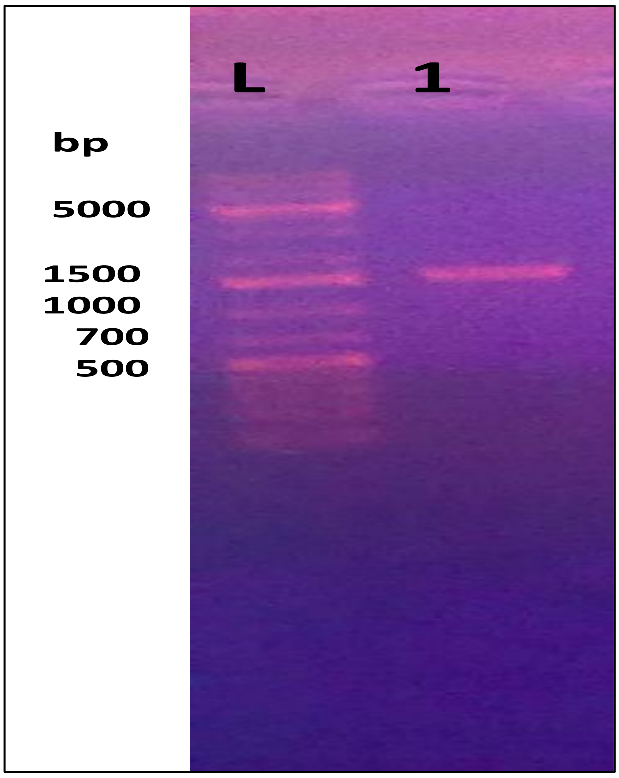

Figure S1. The 16S rRNA gene amplified by polymerase chain reaction (PCR) of the tested *Streptomyces* strain (KM192347). Where, L; GeneRuler^{TM}1 Kb Plus DNA ladder and Lane 1: *Streptomyces* strain KM192347.

TGCTCAGGACGAACGCTGGCGGCGTGCTTAACACATGCAAGTCGAACGATGAACCACTTC
GGTGGGGATTAGTGGCGAACGGGTGAGTAACACGTGGGCAATCTGCCCTGCACTCTGGGA
CAAGCCCTGGAAACGGGGTCTAATACCGGATACTGACCCGCCTGGGCATCCAGGCGGTTC
GAAAGCTCCGGCGGTGCAGGATGAGCCCGCGGCCTATCAGCTTGTTGGTGAGGTAACGGC
TCACCAAGGCGACGACGGGTAGCCGGCCTGAGAGGGCGACCGGCCACACTGGGACTGAGA
CACGGCCCAGACTCCTACGGGAGGCAGCAGTGGGGAATATTGCACAATGGGCGCAAGCCT
GATGCAGCGACGCCGCGTGAGGGATGACGGCCTTCGGGTTGTAAACCTCTTTCAGCAGGG
AAGAAGCGCAAGTGACGGTACCTGCAGAAGAAGCGCCGGCTAACTACGTGCCAGCAGCCG
CGGTAATACGTAGGGCGCGAGCGTTGTCCGGAATTATTGGGCGTAAAGAGCTCGTAGGCG
GCTTGTCGCGTCGGTTGTGAAAGCCCGGGGCTTAACCCCGGGTCTGCAGTCGATACGGGC
AGGCTAGAGTTCGGTAGGGGAGATCGGAATTCCTGGTGTAGCGGTGAAATGCGCAGATAT
CAGGAGGAACACCGGTGGCGAAGGCGGATCTCTGGGCCGATACTGACGCTGAGGAGCGAA
AGCGTGGGGAGCGAACAGGATTAGATACCCTGGTAGTCCACGCCGTAAACGGTGGGCACT
AGGTGTGGGCGACATTCCACGTCGTCCGTGCCGCAGCTAACGCATTAAGTGCCCCGCCTG
GGGAGTACGGCCGCAAGGCTAAAACTCAAAGGAATTGACGGGGGCCCGCACAAGCGGCGG
AGCATGTGGCTTAATTCGACGCAACGCGAAGAACCTTACCAAGGCTTGACATACACCGGA
AACGTCTGGAGACAGGCGCCCCCTTGTGGTCGGTGTACAGGTGGTGCATGGCTGTCGTCA
GCTCGTGTCGTGAGATGTTGGGTTAAGTCCCGCAACGAGCGCAACCCTTGTCCCGTGTTG
CCAGCAAGCCCTTCGGGGTGTTGGGGACTCACGGGAGACCGCCGGGGTCAACTCGGAGGA
AGGTGGGGACGACGTCAAGTCATCATGCCCCTTATGTCTTGGGCTGCACACGTGCTACAA
TGGCCGGTACAATGAGCTGCGATACCGCGAGGTGGAGCGAATCTCAAAAAGCCGGTCTCA
GTTCGGATTGGGGTCTGCAACTCGACCCCATGAAGTCGGAGTCGCTAGTAATCGCAGATC

Figure S2. Sequence of 16S rRNA of *Streptomyces* strain KM192347. eluted PCR product, contains1445bp.
